# Formulating biopharmaceuticals using three-dimensional printing

**DOI:** 10.3389/jpps.2024.12797

**Published:** 2024-03-15

**Authors:** Alistair K. C. Chan, Nehil Ranjitham Gopalakrishnan, Yannick Leandre Traore, Emmanuel A. Ho

**Affiliations:** ^1^ School of Pharmacy, University of Waterloo, Kitchener, ON, Canada; ^2^ Waterloo Institute for Nanotechnology, Waterloo, ON, Canada

**Keywords:** additive manufacturing, drug delivery systems, fused deposition modelling, powder bed fusion, vat photopolymerization

## Abstract

Additive manufacturing, commonly referred to as three-dimensional (3D) printing, has the potential to initiate a paradigm shift in the field of medicine and drug delivery. Ever since the advent of the first-ever United States Food and Drug Administration (US FDA)-approved 3D printed tablet, there has been an increased interest in the application of this technology in drug delivery and biomedical applications. 3D printing brings us one step closer to personalized medicine, hence rendering the “one size fits all” concept in drug dosing obsolete. In this review article, we focus on the recent developments in the field of modified drug delivery systems in which various types of additive manufacturing technologies are applied.

## Graphical Abstract

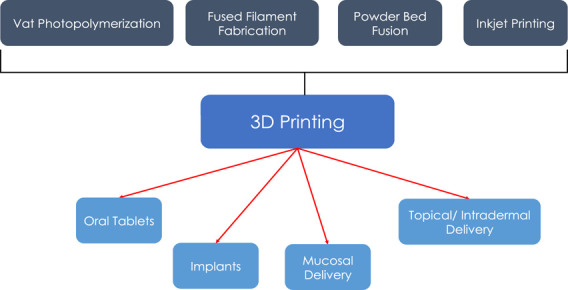

## Introduction

In pharmaceutical formulations, factors such as rate, site, or time of release of the active pharmaceutical ingredient (API) can be altered as desired to create modified release (MR) dosage forms. MR formulations can include delayed release, pulsated release, extended release and more [1]. MR formulations offer various advantages including reduced administration frequency, increased patient compliance, reduced side effects and lengthened duration of action. Ultimately, MR formulations offer better therapeutic outcome while bolstering the quality of life of the patients. Ever since the first-ever United States Food and Drug Administration (U.S. FDA)-approved three dimensional (3D) printed tablet, there has been an increased interest in the application of this technology in drug delivery and biomedical applications. 3D printing enables the rapid prototyping of pharmaceutical products, hence enabling researchers to screen multiple formulations within a short period of time, from which the ideal candidate is selected.

Additive manufacturing, more commonly known as 3D printing, is a process by which 3D objects are printed in a layer-by-layer fashion [2]. The most common types of 3D printing include vat photopolymerization (VPP), fused deposition modelling (FDM), powder bed fusion (PBF), inkjet writing and direct ink writing [3]. In this review, we will focus on the various types of 3D printers used to formulate modified release dosage forms with a brief description on the mechanisms of each subtype of 3D printing.

## An overview of the different types of 3D printing

### Fused deposition modelling (FDM)

FDM is one of the widely explored types of 3D printing, owing to its versatility and simplicity. In fact, FDM-based 3D printing accounts for 80% of indexed literature.

**FIGURE 1:**
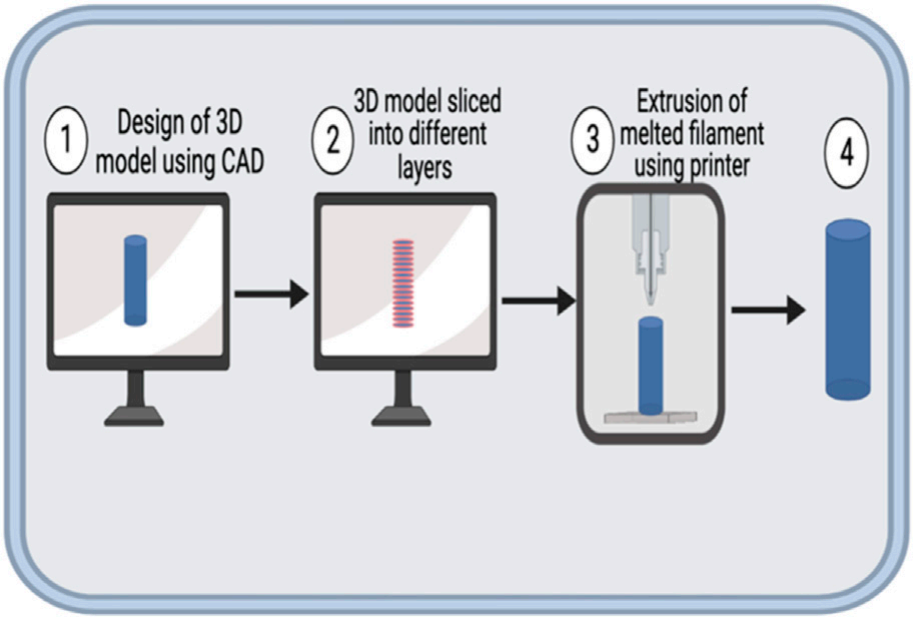
Schematic representation of FDM process. 1. A 3D model of the intended object is generated using a CAD software and saved mostly as a Standard Tesselation Language (STL) file. 2. The 3D model is sliced to layers (outlined in red) using a slicing software. 3. The printer is then used to print the model by extrusion of the melted filament. 4. The finished product might be processed post-printing to impart desired mechanical or aesthetic properties.

The typical FDM process is depicted in Figure 1. Briefly, the polymer for printing, usually in the form of a filament, is fed into a heated liquefier using a pinch roller mechanism [4]. The liquefier is kept at an elevated temperature to facilitate the melting of the polymer [4]. As the polymer is fed through, the melted polymer is pushed through the nozzle at the end of the liquefier [4]. The stepper motors control the movement of the printhead and the temperature-controlled print bed [4]. The designs are usually created using a computer-aided design (CAD) software, which is then transmitted to the printer for printing.

Compared to its counterparts, FDM printing is associated with a diverse assortment of printing materials which are regarded as “biocompatible” or “biodegradable” [5]. For instance, poly (lactic acid) (PLA), a commonly-used polymer in FDM printing, is approved by the FDA as a material for manufacturing medical devices and scaffolds. As a result, it is environmentally-friendly, in that waste materials and leftover filaments can decompose naturally. In addition, FDM printing is cost-effective because through this class of 3D printing, complex 3D objects can be manufactured in short printing times.

FDM has its own demerits that limit its application in the field of pharmaceuticals. First and foremost, most chemical entities are prone to thermal degradation. FDM extrudes filaments at high temperatures, which may be too high for most drugs. As such, most therapeutics associated with FDM should be heat-stable. In addition, heat-labile polymers with low glass transition temperature cannot be employed [6]. This is because such polymers will change from its rigid conformation to a rubbery soft form, which would potentially undermine the polymer’s ability to modulate the rate of drug release, owing to their increased porosity. A potential solution to this would be loading drugs onto the 3D printed structures post-printing. However, drug loading by this method may not achieve high loading efficiency that could otherwise be achieved by extruding the drug and the polymer together.

Another limitation of FDM is the resolution of printing. The size of the nozzle dictates the resolution of the final print. Variables such as the temperature of the heated nozzle and the viscosity of the melted polymer in the heat block can affect the size of the print bead, which in turn affects the print resolution [4]. Other factors such as print speed, die swelling, and road width can also affect the final resolution which has been discussed in detail elsewhere [4].

**FIGURE 2:**
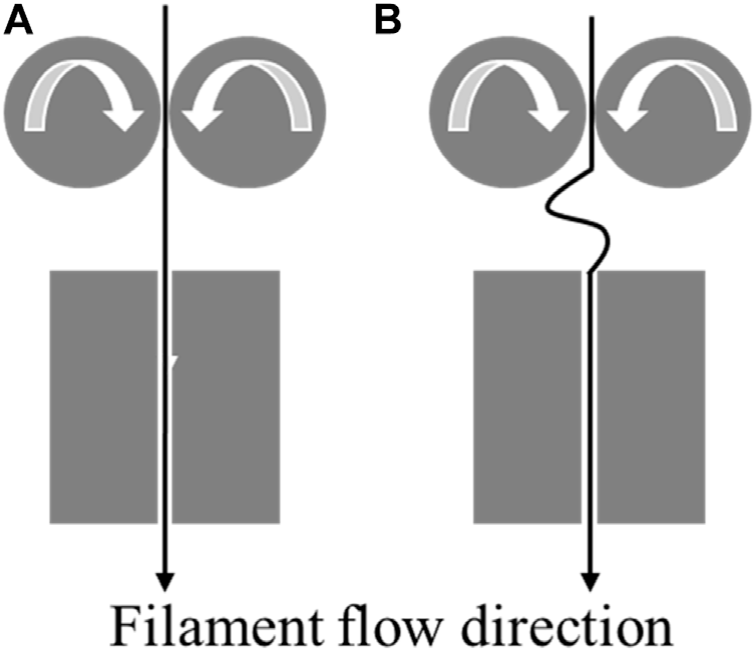
**(A)**: Normal flow of filament through the heated block. **(B)**: Buckling of filament between the heated block and the pinch feed rollers.

Another limitation of FDM is the buckling of filament. Buckling is a common reason for the failure of a print (Figure 2). When the feed rate exceeds a certain limit, the compressive stress from the pinch rollers causes the filament to bend and deform [7], which is very common in flexible filaments.

As a general rule of thumb, polymers employed in consort with FDM printing, such as PLA, polyglycolic acid (PGA), poly (lactic-co-glycolic) acid (PLGA), and poly (caprolactone) (PCL) have low melting temperatures [6]. FDM printing entails the extrusion of molten polymers when the melt extrusion temperature has been reached. As such, the respective melting temperatures of these polymers have to be lower than the respective melt extrusion temperatures of the print systems, which typically range from 90 to 220°C [6].

### Vat photopolymerization (VPP)

VPP is another widely used class of additive manufacturing. Benefits of this class of 3D printing include enhanced printing resolution and increased print volume. The three most common sub-types of vat photopolymerization include stereolithography (SLA), digital light processing (DLP) vat photopolymerization and two-photon polymerization [3].

#### Stereolithography

**FIGURE 3:**
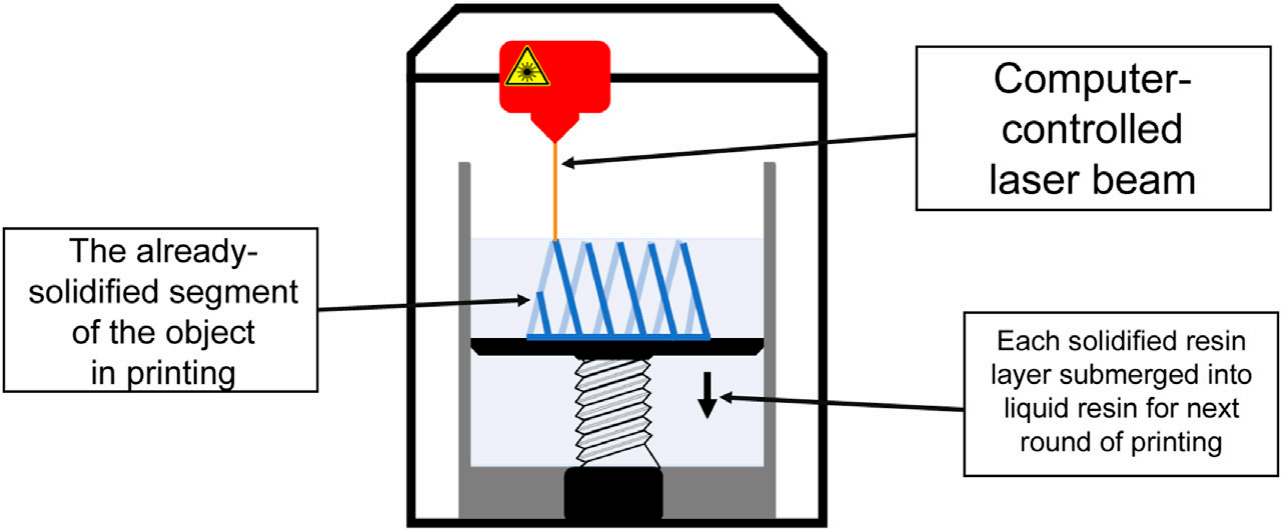
A schematic of SLA printing. Each layer of the desired object is printed when a computer-controlled laser beam is projected onto the liquid resin surface in the desired pattern, leading to solidification. Each of the formed layer is then submerged into liquid resin and new resin is allowed to solidify atop the surface and the process is repeated until the entire desired object has been printed.

In SLA, polymerization of the liquid resin is achieved by a computer-controlled laser beam which is projected onto the resin surface in the desired pattern (Figure 3). This leads to the solidification of the resin in the shape of the pattern. The formed layer is then moved down the pool of liquid resin and new resin is allowed to solidify atop the surface and the process is repeated [8] (Figure 3). One of the major limitations of SLA is the limited amount of polymers available for this method, because only photocurable materials can be employed [8].

#### Digital light processing (DLP) vat photopolymerization

In DLP, the light is reflected by a digital micromirror device (DMD) onto the photo-sensitive polymer. Rapid toggling of DMDs is employed to direct the light onto desired coordinates on the print surface [9]. DLP is a fast and reliable method. However, it is again limited by the number of compatible polymers. Methods of making materials DLP-compatible include the addition of a photosensitive excipient and chemical modifications. Unfortunately, this makes them toxic and unfit for pharmaceutical or medical applications [10]. To this date, there has been no FDA-approved resin [6].

#### Two-photon polymerization (TPP)

TPP utilizes two lasers that shine at a specific coordinate in the build volume, hence initiating polymerization at that specific coordinate [3]. A pitfall for two-photon polymerization, is that it is relatively expensive which in turn limits its application, especially for mass production [3].

Polymers that are typically employed in VPP include gelatin methacrylate (GelMA), polyethylene glycol diacrylate (PEGDA), and hyaluronic acid methyacrylate (HAMA), all of which are essentially the acrylated counterparts of their respective precursors [6]. In addition to being photosensitive, these materials should also be of high fluidity, lest the misalignment of the different layers of the print product that are being printed atop each other. However, recently, resin of high viscosity have been manufactured in a bid to enhance the mechanical properties of the VPP-printed product [11].

### Powder bed fusion (PBF)

**FIGURE 4:**
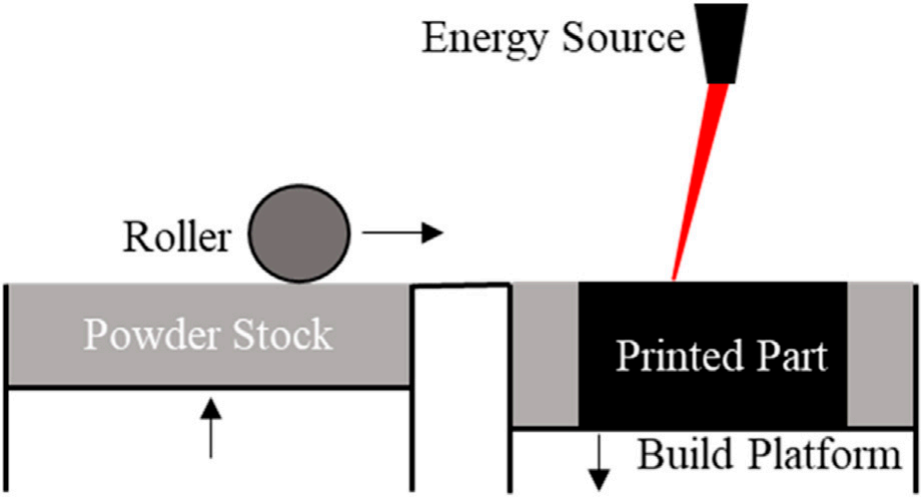
A schematic of PBF printing. Each layer of the desired object is printed when an energy source, either a laser or an electron beam, fuses the powder on the build platform. A new layer of powder is spread, using a roller, from the powder stock across the printed layer and the process is repeated until the entire desired object has been printed.

PBF is another type of 3D printing where powdered material is spread in layers followed by melting and sintering, either by a laser or an electron beam (Figure 4). In general, since the size of the powder dictates the printing resolution, the thickness of every layer in the printed objects are designed to be smaller than the size of its precursor powder, and typically falls into the range of 15–300 µm [6].

### Inkjet printing

Inkjet printing is another form of additive manufacturing that has recently been explored for its application in the pharmaceutical industry. Inkjet printing entails two subtypes, namely continuous inkjet (CIJ) and drop-on-demand (DOD) printing [12].

#### Continuous inkjet (CIJ)

In CIJ, as the name suggests, ink is continuously passed through the nozzle which is then broken into droplets. These droplets are then deflected by a charged plate onto the print surface. Unused droplets are collected and recycled for subsequent printing [12].

#### Drop-on-demand (DOD) printing

In DOD, ink droplets are generated as needed and directed onto the print surface and allowed to dry.

CIJ is a relatively faster process and has a lower resolution than that of DOD. Additionally, due to the recycling of ink in CIJ, there is a chance of contamination of ink thereby affecting the printing and drying properties. DOD on the other hand yields better resolution than CIJ but could suffer nozzle clogging [12]. However, inkjet printing is typically associated with high manufacturing costs [6].

## 3D printed oral tablets

Oral tablets are solid unit dosage forms that contain one or more APIs. Despite advances in the field of drug delivery, oral tablets remain one of the most used dosage forms, as they are cost-effective and user-friendly. 3D printing is an alternate means for manufacturing tablets. In August 2015, the U.S. FDA approved the first-ever 3D printed tablet, SPRITAM^®^. SPRITAM^®^ was reported to be able to accommodate a higher drug loading of up to 1,000 mg of therapeutics, in addition to instant release by disintegrating roughly 10 times faster than what conventional tablets would [5]. This has attracted a lot of attention in the application of 3D printing technologies in pharmaceuticals. Spritam^®^ is printed using ZipDose^®^ technology, where a layer of the powder blend containing levetiracetam and the excipients are spread on a surface [5]. A binding liquid is then sprayed on the powder blend [5]. These steps are repeated until the desired dose is obtained [5]. In contrast to conventional means of tablet manufacturing, a wider range of powdered materials are compatible, should the right binding material be ascertained [5]. In addition, even heat-labile drugs can be formulated into tablets via such means, as opposed to FDM printing which as aforementioned is only compatible with heat-stable drugs. This is because inkjet printing can be carried out at room temperatures [5].

One of the first publications incorporating FDM for the manufacturing of tablets was published by Goyanes et al. [13]. The group extruded PLA as their print material. Fluorescein was loaded onto the filaments as a model drug by soaking PLA filaments in ethanol-dissolved fluorescein. The filaments were then quantified for the amount of loaded fluorescein and tablets with different concentrations of fluorescein were printed by varying the infill densities, thus highlighting the versatility of FDM in the dosing of pharmaceuticals. The 3D printed tablets were shown to have an extended drug release profile lasting for up to 10 h.

The same research group studied how the print geometry of the 3D printed tablets would modulate the release profile of payload [14]. Paracetamol (acetaminophen) was mixed with PLA as the model drug and extruded via hot-melt extrusion (HME). HME was chosen over drug solution soaking owing to the higher drug loading capacity of HME. The drug-loaded PLA filament was then used to 3D print tablets of different geometries such as cube, sphere, pyramid, cylinder, and torus-shaped. FDM has the advantage of being able to print some complex geometries that might not be achievable without the need for support materials as in the case of other types of printers. It unveiled that, pyramid, the shape with the highest surface area/volume ratio, had the fastest drug release while sphere, with the lowest ratio, had the most-sustained release for up to 12 h. This demonstrates that the geometry of the printed dosage forms can be altered to tune the drug release rates.

Okwuosa et al. were able to print a delayed-release tablet bearing a core-shell structure by employing two different polymers [15]. This was achieved by using a multi-extruder printer, which prints both the shell and core simultaneously. The core was composed of a blend of polyvinyl pyrrolidine (PVP) and theophylline as the API, alongside other additives, whilst the shell was fabricated using Eudragit L100-55, a methacrylic acid–ethyl acrylate copolymer which is widely used in enteric coating.

Gaisford et al. used a customized SLA printer to create a multi-layered tablet, where each of the layers contain a different drug [16]. The six drugs were prednisone, naproxen, acetaminophen, caffeine, chloramphenicol and acetylsalicylic acid (ASA). The tablet was printed such that drugs with higher water solubility were printed in the inner layers while their counterparts were printed in the outer layers. Printing can be halted as desired, allowing the build platform to raise in order to facilitate the switching of the different resin trays such that the aforementioned multi-layered tablet can be manufactured. The authors also evaluated the influence of the tablet geometry on the drug release profiles up to 20 h. This study demonstrated the potential of 3D printing in manufacturing custom medication combinations for patients on multiple drug therapies. A modified release paracetamol (or acetaminophen) and 4-Amino salicylic acid (4-ASA) tablets were fabricated by Wang et al. using a commercial SLA printer [17]. The authors were able to modify the release of the drug by adjusting the ratio of polyethylene glycol (PEG) and poly (ethylene glycol) diacrylate (PEGDA) to achieve over 10 h of drug release.

Fina et al. were able to successfully use selective laser sintering (SLS) to formulate paracetamol tablets. The tablets were made of either Kollicoat^®^ or Eudragit^®^ in varying amounts and 3% Candurin^®^ Gold Sheen as the absorbent [18]. The use of different polymers and different polymer to drug ratios were shown to affect the release pattern, with Eudragit^®^ tablets displaying up to 12 h of release. Allahham et al. used a similar protocol wherein they printed Ondansetron tablets using SLS [19]. Ondansetron, an anti-emetic medication, was formulated as a cyclodextrin inclusion complex and mixed with multiple ratios of Kollidol and mannitol with 3% Candurin^®^ Gold Sheen. Ondansetron is widely marketed as an orally disintegrating tablet and the authors were able to formulate a tablet with a similar dissolution profile, where the 3D printed tablets displayed over 80% drug release in the first 5 minutes. These formulations have further demonstrated the versatility of 3D printing and the effects of the excipients used.

Inkjet printing has also been explored in the manufacturing of tablets. Fenofibrate tablets were 3D printed by Kyobula et al., using beeswax as the base [20]. The authors investigated the effects of initial drug loading and the surface geometry on the drug release rate. The tablets were printed either as a solid tablet or in a honeycomb pattern, using a piezoelectric inkjet printer (PiXDRO LP50). Tablets printed with the honeycomb pattern showed faster drug release owing to its higher surface area. Additionally, high drug loading was shown to slow down drug release, which could be a result of the drug in its crystalline form. The formulated tablets showed up to 12 h of drug release. Clark et al. used inkjet printing to fabricate ropinirole tablets, a medication used in the treatment of Parkinson’s disease [21]. The authors used inkjet printing coupled with ultraviolet (UV) photocuring to formulate the tablets. The ink was prepared by solubilizing ropinirole in PEGDA containing Irgacure 2,959 as the photo-initiator. The print was carried out on poly (ethylene terephthalate) film in a nitrogen purged chamber. The photocuring was accomplished using a UV Light emitting diode (LED) lamp that was mounted onto the extruder. The tablets exhibited drug release for up to 4 h, with almost 60% of the loaded drug being released in the first hour owing to the highly solubility of the drug’s salt form.

## 3D printed implants

Although the oral route has been the primary route of drug administration for years due to its low cost and ease in administration, chronic administration of oral tablets has typically been associated with low patient compliance. Additionally, APIs administered orally undergo first-pass metabolism at the liver, resulting in lower concentrations at their sites of action. Administration of a higher dose to compensate for the first-pass metabolism could lead to unwanted side effects. These demerits can potentially be overcome via the local administration of a dosage form that would elute drugs over extended periods of time. As a result, this would help reduce adverse reactions, increase compliance, thereby enhancing clinical outcomes. Implants are delivery systems where the API is loaded usually in a polymeric carrier that is subsequently implanted in the system by a healthcare professional. The different types of polymers used in the manufacturing of implants and the mechanism of drug release are discussed in detail elsewhere [22]. In this section we will focus on the various drug eluting implants that are fabricated using additive manufacturing technology.

Stewart et al. created an implantable drug delivery system that was fabricated using FDM from a combination of PLA and poly (vinyl alcohol) (PVA) filaments [23]. These reservoir-type implants were made of PLA, with PVA windows of varying numbers and dimensions embedded onto the implant to control the rate of drug release. Drug release of these implants were then assessed in agitated release media and agarose gel models. Based on the implant design, the authors demonstrated drug release of up to 25 and 40 days in the agitated media and the agarose gel model, respectively. The release profile of the implants was substantially prolonged up to 300 days when they were coated with a PCL mixture. This demonstrated the potential of coating a thin film on the surface of implants as a further means to modulate drug release and could potentially be used in the treatment of long-term chronic conditions. As a subcutaneous implant, it has to be surgically implanted into patients, meaning that it has to undergo prior sterilization, lest they lead to infection and even toxic shock syndrome which is potentially fatal. However, the authors made no remarks about the sterilization procedures that would be ideal for this implant.

A modified β-lactoglobulin or the JB Protein was used by Zhao et al. to fabricate a cervical implant to prevent human papilloma virus (HPV) infection [24]. The authors utilized low-temperature deposition modelling (LDM), wherein a polymer solution, thermoplastic polyurethane (TPU) in this case, is extruded through a nozzle at a temperature between −30°C and −40°C. This ensures that the TPU is solidified rapidly. It is then placed in a freeze dryer to remove any solvent, thereby creating micropores that will in turn act as a reservoir for the drug molecules. The authors were able to achieve a release of up to 20 h, and the pores are regulated such that both the loading and release of anti-HPV proteins can be modulated.

Heinl et al. employed selective electron beam melting (SEBM) and a titanium alloy, Ti-6Al-4V, to create cellular bone implants for orthopaedic purposes [25]. In SEBM, an electron laser is used to melt layers of powder, resulting in porous materials with well-defined cellular structures. Two structures, namely, diamond and hatched structures, were fabricated by scanning the titanium alloy powder in a layer-by-layer fashion. The generated samples were then etched using 37% hydrochloric acid at 50°C for 90 min in an argon atmosphere. They were then soaked in 10 M sodium hydroxide for 24 h at 60°C, after which they were washed and dried. These steps increased the amount of hydroxyl groups on the surface of the prints, which is attributed to the improved fixation of the implant in the surrounding bones, as well as the enhanced long-term stability of the implant. The implant was shown to possess comparable physical characteristics of certain human bones. In addition, SEBM can be used to adjust the mechanical properties by modulating the porosity of the print.

## 3D printed formulations for mucosal delivery

The mucosal membrane, or mucosa, are the cells that line the inner surface of body cavities such as the gastrointestinal, vaginal and respiratory tract. They are characterized by epithelial cells covered in a layer of mucus, a thick, viscous secretion that is composed of water, electrolytes and proteins such as mucin [26]. They serve to protect the underlying epithelial cells, aid in the movement of substances along the tracts they cover, and retain moisture in the epithelium [26]. From a pharmaceutical point of view, mucoadhesion is described as the state in which a drug delivery system adheres to a mucosal membrane using interfacial forces, and release drugs over extended periods of time [27]. Various mucoadhesive formulations such as oral mucoadhesive tablets, gels and vaginal films were successfully formulated for extended drug release [28–34]. These formulations have been used for both local and systemic release to bypass first-pass metabolism as discussed earlier, thereby increasing the bioavailability of the drug molecules. These bioadhesive formulations were made using different blends of polymers composed of hydroxypropyl methylcellulose (HPMC), carbopols, carboxymethylcellulose and other polymers as needed.

Mucoadhesive formulations are optimized to obtain the desired retention time and release rate by adjusting the ratio of the constituent materials in the polymer blend. Tagami et el., used a commercial 3D printer to fabricate a vaginal suppository for the controlled release of progesterone [35]. The authors used PVA to create the shells of the suppositories with holes on the walls of the shell. The size and locations of the pores in the shell were varied to modulate the release of progesterone from the shells. The authors also formulated a multi-layered shell to achieve a pulsatile release of the drug.

**FIGURE 5:**
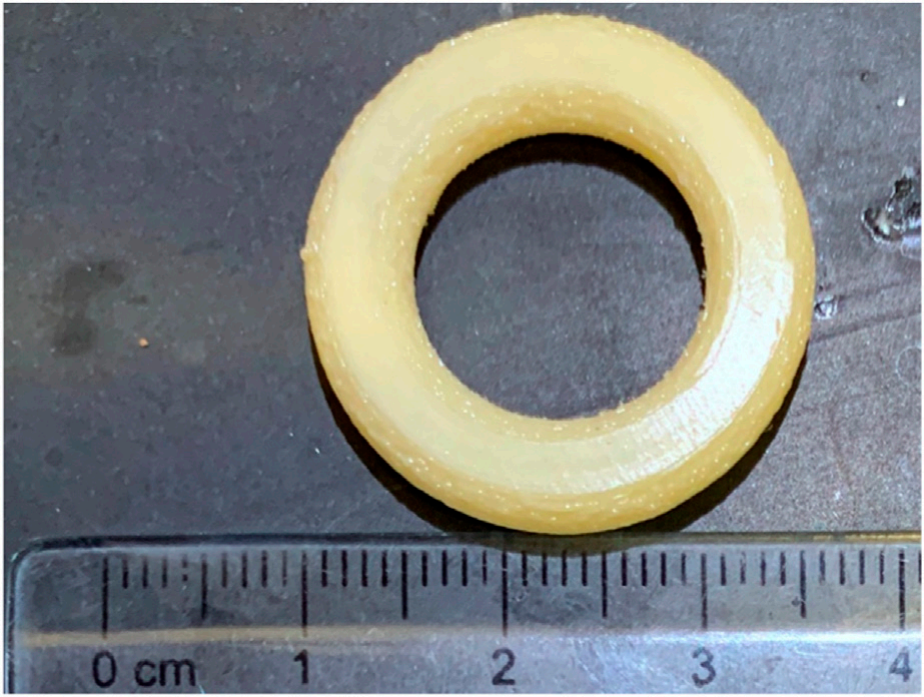
Prototype of a macaque-sized IVR printed with a custom-built FDM printer built by Laboratory for Drug Delivery and Biomaterials, Ho Research Group, University of Waterloo.

Intravaginal rings (IVR) are formulations developed to deliver drugs locally in the vaginal tract. IVR were first explored as a form of drug delivery system to provide a more discrete and long-acting form of contraceptive for women [36]. NuvaRing^®^, Femring^®^ and Estring^®^ are some of the FDA approved contraceptive rings currently marketed [37]. More recently, Annovera^®^, a year-round contraceptive vaginal ring was approved by FDA in 2018 making it one of the longest acting contraceptive IVR [38]. With IVRs gaining popularity as a contraceptive, 3D printing is explored to fabricate IVRs for not just contraception, but also other biomedical applications. For instance, 17β-Estradiol IVR was one of the first IVR formulations to treat urogenital atrophy in women [39]. IVRs have also been employed for the delivery of microbicides, therapeutics that are applied topically to prevent sexually transmitted infections during sexual intercourse [40]. A prototype of an intravaginal ring as printed with FDM is shown in Figure 5.

Chen et al. recently developed, by virtue of FDM printing, a novel reservoir IVR that can provide the tunable, controlled release of hydroxychloroquine (HCQ), immunoglobulin G (IgG) and enveloped glycoprotein GP120 (GP120) fragment, and coumarin 6-encapsulated PLGA-PEG nanoparticles, to potentially offer a comprehensive and potentially synergistic protection against HIV infection in the female vaginal tract [41]. Briefly, HPMC containing HCQ was loaded onto HP-60D-35 IVR segments printed using FDM to ascertain the effect of the thickness of the rate-controlling membranes on the release kinetics of hydrophilic small molecules [41]. It was unveiled that the thickness of the rate-controlling membranes can simply be modulated with high reproducibility, in that there exists a linear correlation between the thickness of the rate-controlling membranes and the number of printing shells [41]. The study demonstrated that the release of HCQ above a clinically effective concentration from such fragments showed tunable zero-order release kinetics over 14 days, which is approximately 7 times the duration in some previous literature [41]. Besides, it is one of the first reports where the sustained release of hydrophilic APIs can be achieved. On the other hand, HPMC containing IgG and GP120, and PLGA-PEG nanoparticles were loaded onto ATPU-75A IVR segments printed using FDM to discern how the varying segment geometries, as dictated by different interior fill density and printing patterns, would affect the release of macromolecules and nanoparticles [41]. It was revealed that, amongst the ATPU-75A IVR segments with the grid pattern that confers themselves uniform porosity, larger pore sizes owing to lower interior fill densities would lead to faster release rate [41]. Although nearly zero-order release kinetics for up to 2 weeks were achieved for both IgG and GP120, the rate of release is inversely related to the size/molecular weight of the payload [41]. The effect of printing pattern on the release kinetics of PLGA-PEG nanoparticles was also scrutinized, and it was revealed that regardless of the interior fill densities, grid printing pattern with more uniform porosity facilitates a more consistent release of nanoparticles, as opposed to a triangular printing pattern with more irregular porosity [41]. All in all, this article is the first to demonstrate superiority, simplicity, feasibility, and versatility of FDM printing in developing reservoir IVR fragments that can provide constant levels of therapeutics of different chemical classes, namely hydrophilic small molecules, macromolecules and polymeric nanoparticles over an extended period of time.

Fu et al., developed novel shaped intravaginal implants for the delivery of progesterone [42]. The authors used a combination of PLA and PCL, with Tween 80 and progesterone dispersed in PEG 4000 to create filaments for 3D printing. The filaments were then used in a FDM printer to fabricate “O,” “Y,” and “M”-shaped vaginal implants (Figure 4). These implants exhibited an extended-release pattern over a course of 7 days, with the “O” shaped implant showing the highest release. Despite the “O” and “Y” shaped implant having the same surface area/volume ratio, the fast and high release of progesterone from the “O” implant was attributed to its unique shape. These rings also showed an initial burst release followed by a slow and steady release which could be desirable from a therapeutic perspective.

A clotrimazole-loaded, matrix type IVR was developed by Tiboni et al. using TPU. Clotrimazole was dispersed onto the surface of the TPU pellets using castor oil, which was then extruded into a FDM filament using a hot melt extruder. The rings were then evaluated for their release and antimicrobial efficacy against *Candida albicans.* The ring was demonstrated to be able to release clotrimazole for at least 7 days, with the concentrations above the minimum inhibitory concentration.

## 3D printed formulations for topical and intradermal drug delivery

Topical drug delivery refers to the route of drug administration through which formulations are applied to predominantly the cutaneous membrane, but also the respective mucosa of the eye, nose, vagina and even mouth, with the intent of achieving therapeutic concentrations of medication in the concerned areas to treat local diseases [43]. This section would exclusively be dedicated to the former case of topical drug delivery, more specifically, intradermal drug delivery.

The aim of cutaneous topical drug delivery is simply to deliver therapeutic concentrations of medication to local tissues [44]. On the other hand, transdermal drug delivery aims at delivering high concentrations of therapeutics into the dermis, allowing the drug to be taken into the systemic circulation [44].

A great many efforts have been invested heavily in topical drug delivery systems, be it 3D printed or not, for it is more meritorious than conventional routes of drug administration, namely oral and parenteral [45]. From the pharmacodynamic perspective, the intradermally-administered drugs would bypass first-pass metabolism in the liver [3]. From a practical point of view, intradermal drug delivery systems have been reported to result in higher patient compliance owing to their relative ease in administration using non-invasive, pain-free techniques [45].

As such, this section aims to highlight how 3D printing can be employed to tackle delivery challenges associated with topical delivery.

**FIGURE 6:**
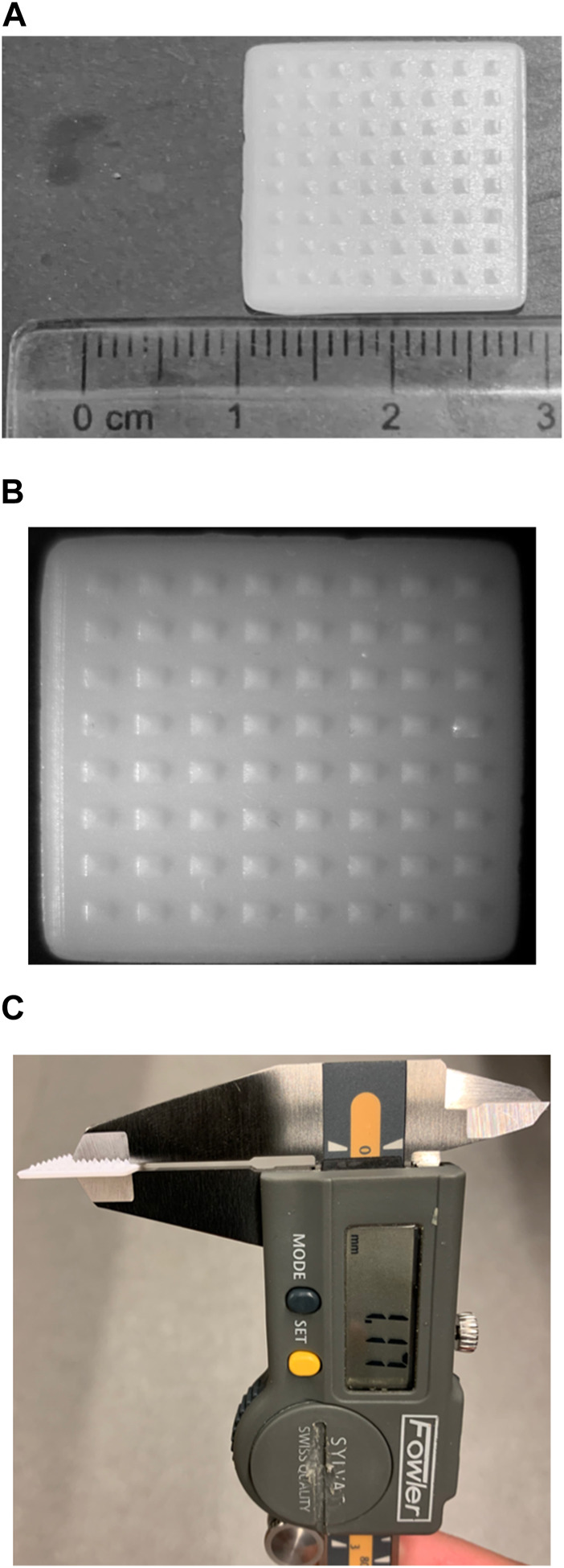
A prototype of a microneedle patch printed with a vat photopolymerization printer built by Laboratory for Drug Delivery and Biomaterials, Ho Research Group, University of Waterloo. **(A)** Depicts the length and width of a prototype microneedle patch **(B)** depicts the prototype microneedle patch, taken with a OMAX 3.5X-90X Digital Trinocular Stereo Microscope attached with 0.5X Reduction Lens for Microscope Camera and 144 LED Light, courtesy of Drug Delivery and Pharmaceutical Nanotechnology Laboratory, Foldvari Research Group, University of Waterloo **(C)** thickness of the patch as determined using a digital caliper.

Microneedle arrays consist of needles with a length of roughly 25–2000 μm [46]. All in all, microneedles are robust enough to penetrate the resilient stratum corneum, therefore allowing the delivery of therapeutics to the cutaneous layers [46]. Yet at the same time, microneedles do not stimulate the nerve fibers and blood vessels in the dermal layer, hence conferring themselves their signature “pain-free” characteristic [46]. A microneedle patch prototype printed via vat photopolymerization is shown in Figure 6, which demonstrates the ability of vat photopolymerization printers to print objects of fine details. Table 1 summarizes how different types of 3D printing enable the fabrication of microneedles for various intradermal applications.

**TABLE 1:** Summary of the different microneedles made using different types of 3D printing for different biomedical applications topically.

Method	Biomedical Application(s)	Scaffold Material(s)	Scaffold(s)	References
FDM	Glucose-responsive insulin, transdermal drug delivery, pain management	Alginate with hydroxyapatite as an additive, PLA, PDMS, multiwalled carbon nanotubes	Microneedle patch, biodegradable polymeric microneedle arrays, microheater-integrated microneedle patch	[46–50]
SLA	Transdermal drug delivery, transdermal delivery of high weight antibiotics, trans/intradermal delivery of insulin, microencapsulated cell extrusion, transdermal delivery of cisplatin for anticancer therapy of skin tumours	PVA, PLA, Polyester resin, Acrylonitrile butadiene styrene (ABS), PVP, IP-S photoresist, Class I acrylic resin Dental SG, Carboxymethyl cellulose, methacrylate, Formslab Castable Resin, Formlabs class IIa biocompatible resin, Soluplus^®^	Solid microneedle, polymeric microneedles, hollow microneedles cojoined with reservoir void, hollow microneedles, microchannel-microneedle platforms, polymeric microneedle- microelectromechanical system	[51–58], [59-62]
DLP	Transdermal drug delivery, transdermal delivery of diclofenac for treatment of trigger finger, transdermal delivery of Acetyl-hexapeptide 3 for anti-wrinkling	PEGDA, PVA and sucrose, Kudo3D Inc. 3DM Castable resin, mixtures of PEGDA and vinyl pyrrolidon, EnvisionTEC Inc. Photocurable Eglass resin	Backward-facing barbs entrenched microneedle arrays ‘tanto blade’-inspired polymeric microneedle arrays, personalized microneedle splint on curved surfaces, personalized polymeric microneedles based on 3D scanned face model, limpet tooth-inspired microneedles, Hydrogel-forming Microneedles	[63-67]
Liquid Crystal Display	Transdermal delivery of octreotide acetate (peptide)	Nextdent Ortho Rigid resin	Hollow microneedle with drug reservoir	[68]
Two-Photon polymerisation	Transdermal delivery of cabotegravir and ibuprofen, multicomponent cutaneous vaccination, perforation of human round window membrane as a physical enhancement strategy for inner ear delivery, aspiration of perilymph across the human round window membrane for sampling	PVP and PVA 50 K, Gantrez^®^ S-97, PEG 10,000 Da and Na_2_CO_3_, carboxymethylcellulose, PVP, PVA, crystal Si (100) wafer, photoresist IP-S	Dissolving polymeric microneedle array, hydrogel-forming microneedle array, solid microneedle array, hollow microneedle	[69-73]

3D-printed pharmaceutical products are employed for the purpose of wound-healing. For instance, Singh et al, using melt-extrusion-based 3D printing, were able to fabricate bandage-like wound coverings. The semi-crystalline PLA was selected as the base polymer which was subsequently coated with the plasticizer PEGs of varying molecular weights (0.4 kDa, 6 kDa or 20 kDa) and loaded with neomycin, an aminoglycoside antibiotic which hampers topical infection using a soaking method in which the neomycin passively diffuses into the soaked and swollen PLA molecules [47]. Owing to the large pores (0.1*0.4 mm) they possess, such mats not only provide a large surface area crucial to cell and platelet adhesion which initiates clot formation and consequently the wound-healing cascade but also confers permeability to congealed plasma, hence, speeding up healing [47]. Despite the fact that the different molecular weights of PEG coated onto the coverings play no role in altering the duration and mechanism (first-order release kinetics for the first 20 h, followed by Fickian diffusion-controlled release for the remainder of drug release) of neomycin release, there seems to be an inversely proportional relationship between the molecular weight of coated PEG and drug loading, as inferred from the trend where the absolute amount of drug released decreases with increasing molecular weights of PEG [47].

In another study, Derakhshandeh et al. produced smart, individualized bandages encompassing hollow, miniaturized needle arrays (MNA) that were printed using FDM to deliver vascular endothelial growth factor (VEGF) and other therapeutics with independent temporal profiles to the deeper layers of the wound bed [48]. The integrated bandage consists of 2 modules, the reusable module composed of the drug reservoirs, micropumps and power source and the disposable module comprising the microchannel arrays micromolded into the bandage made of Polydimethylsiloxane (PDMS) and MNA islands, both of which were bonded together through silanization [48]. The MNAs were designed to have spacings of 1.5–3 mm between the different needles, base sizes of 0.5–1.5 mm, opening diameters of 0.2–0.5 mm and needle lengths ranging from 0.8 to 3 mm [48]. Needles with a length of 2 mm were ultimately selected for delivery, having taken into account the thickness of the dermis and the epidermis of the human skin [48]. A smartphone application was developed to interface with the modules to envision remote regulation of topical delivery of therapeutics [48]. Human umbilical vein endothelial cells (HUVEC) migration assay revealed that the migration of HUVEC cells incubated with culture media containing MNA-delivered VEGF have comparable migration rates as the positive control group bathing in VEGF directly whilst the cells that are incubated in culture media containing conventional topically-delivered VEGF have minimal migration rates [48]. Animal studies involving B6.BKS (D)-Leprdb/J mice revealed by the end of the 19-day study that the scars in mice administered VEGF via MNA reached an average of 95% closure while those of mice administered with VEGF via ordinary topical means and no VEGF (negative control) achieved 55% and 40% closure, respectively [48]. Both the *in vitro* and *in vivo* tests highlighted the superiority of MNA over conventional topical administrations of VEGF in the context of wound healing owing to the fact that the former could administer medication to the deeper parts of the wound bed as iterated.

However, FDM is not without its pitfalls, in the context of intradermal delivery. Goyanes et al. successfully synthesized individually tailored anti-acne nose-shaped masks based on the respective 3D models of individuals’ nose morphologies [49]. Flex EcoPLA™ fibers were loaded with salicylic acid via hot extrusion for 3D printing [49]. The FLPA-salicylic acid filaments, bearing a uniform diameter of 1.67 ± 0.16 mm, have a drug loading of 0.63 ± 0.10% w/w, which is lower than the theoretical loading of 2% w/w. It was postulated, with the support of thermogravimetric analysis data, that such a discrepancy is attributed to the degradation of salicylic acid as a result of the high extruding temperature (190°C) [49]. To make things worse, the mean drug loading in the products 3-D printed at 230°C using was further reduced to 0.35 ± 0.01% w/w, thus highlighting FDM-associated thermal degradation of therapeutics as one of its downsides [49].

Apart from FDM, other variations of 3D printing were also employed for the fabrication of devices for sustained topical delivery. Crosslinked poly (acrylic acid) (PAA) and micellar Pluronic F127 (F127) hydrogels embedded with cellulose nanocrystals (CNC) were synthesized by photoinitiating the photopolymerization of PAA with the technique of DLP. The material was subsequently post-cured under UV light and used as matrices for the delivery of nitric oxide (NO) donor S-nitrosoglutathione to attain local vasodilation, wound healing and analgesia [50]. Data from cryo-TEM revealed that the micellar packing morphology of F127 was preserved post-photopolymerization [50]. PAA/F127 hydrogels were able to bear a maximum of 0.25M Pa stress at 50% with high compressive recoverability [50]. PAA/F127 hydrogels loaded with 0.25% (w/w) of CNC have the highest Young’s Modulus when hydrated and therefore have the highest biomedical relevance owing to its better mechanical properties [50]. The release of NO is bimodal, with a loaded NO-dependent fast-step which lasts until 60 min since hydration and a slow step which has comparable rates across all hydrogels loaded with varying amounts of NO [50]. The former step was postulated to be related to diffusion of NO in proximity to the hydrogel/water interface and hence is NO-dependent whilst the latter step is associated with the rate of diffusion of NO embedded inside the hydrogel matrix and hence has nothing to do with the concentration of NO [50]. In a nutshell, such a means of 3D printing was established to print hydrogels which faithfully replicate the features of the 3D models capturing individual’s face morphologies, hence casting light to the possibility of using such hydrogels for multi-purpose topical treatments [50].

Cylindrical (1.9 cm or 4.0 cm radius × 2 mm height) cryptotanshinone-loaded niosomal hydrogels (CPT-NH) were 3D-printed via a semi-solid extrusion method for acne treatment [51]. The rationale for employing 3D printing is that, by incorporating varying amounts of therapeutics in the mixture to be 3D-printed, the dose of such printed hydrogels could be easily tailored to suit every individual’s demands [51]. In short, the reverse evaporation-synthesized CPT-loaded niosomes were first and foremost screened using Quality-By-Design for the optimal combination of particle size and encapsulation efficiency [51]. The optimized formulation of CPT-loaded niosomes were then added dropwise to a homogenous mixture of hydrogel consisting of sodium polyacrylate 700, glycerin and aluminum glycinate serving as the base material of the hydrogel, the moisturizer, and crosslinker, respectively, which was subsequently loaded onto the 3D printer for semi-solid extrusion printing [51]. CPT-NH has pH similar to that of the skin and hence makes an excellent candidate for topical medication, alongside the fact that the bio-adhesive properties of CPT-loaded niosomes remain unaltered after 3D printing [51]. *In vitro* release revealed that CPT was encapsulated in niosomes, thus suggesting that the structure of niosomes was preserved amidst the different treatments related to the 3D printing of hydrogels. CPT were released in the fashion as depicted in the Korsmeyer-Peppas model [51]. Whilst CPT-NH induced no irritation in the skin, it was demonstrated to be efficacious in hampering the development of acne [51]. All in all, 3D printing was validated to produce hydrogels of consistent sizes and CPT doses and as such is a viable means through which the various dimensions of CPT-NHs are customized for personalized treatment of acne [51].

## Discussion

Tablets being one of the most used dosage forms, various authors have tried various approaches in creating different types of tablets. Many drug molecules tend to be heat labile or photosensitive or both. Overcoming this could be a major obstacle if FDM is employed. Soaking the printed tablet in a drug solution could be used for heat sensitive drugs but the dose loaded might not be sufficient to reach therapeutic concentrations or sustain the concentration for longer periods of time. Alternatively, a reservoir type oral dosage form could be fabricated and the drug in solution or powder form could be filled within the 3D printed walls of the tablet. The design parameters could be adjusted to achieve the desired release. Inkjet printing and other methods of printing requires a photo-initiator and other excipients which might not be desirable due to its potential side effects. Many photo-initiators have been shown to be toxic to liver and kidneys in animal models and cell lines [52]. Some photo-initiators have been shown to be biocompatible, but more research needs to be done to explore the long term effects in biological systems [52, 53]. Although post processing could be used to eliminate any toxic materials, most require soaking in solvents which might cause leaching of drugs thereby reducing the true dosage. Another important consideration with the use of various polymers is the environmental impact the waste materials could potentially have.

One of the major necessities for implantable devices is sterility. Most 3D printing methods do not employ sterile techniques and hence the final product will need to be sterilized. Some of the most commonly used sterilization techniques employed include heat, UV or ethylene oxide sterilization. A study carried out by the Canadian Nosocomial Infection Surveillance Program over a period of 10 years estimates the average number of surgical site infections to be between 26,000–65,000 every year [54]. This puts the patient at a risk of septicemia and septic shock which could lead to death. Hence care must be taken to ensure that the 3D printed implantable devices are completely sterilizable. Another major concern with regards to implants is the development of biofilm. Treatment of biofilms requires high doses of antibiotics which might cause various side effects to the patient. Approaches like antibiotic coating of the implants or surface modification of the polymeric implants could be used to minimise the risk of biofilms [55].

Mucosal drug delivery helps deliver the drug molecule close to the targeted site thereby reducing the dose required and hence reducing number of side effects. Not all mucosal surfaces in the human body are identical. With some membranes being more permeable than the others, formulation development should take this into consideration to determine dosing and drug release rate. Acidic drugs could potentially cause mucosal membrane degradation over chronic therapies and care must be taken to protect the mucosal layer. Additionally, mucosal layers have high concentration of enzymes which might be able to degrade the drug molecule or the formulation itself.

Whilst topical drug delivery may sound superior to other routes of delivery, it is not immune to delivery challenges just as its counterparts are not, if not more prone. One of the most prominent delivery challenges is none other than the physiological barriers of the body, which although shields the human body from the invasion of foreign harmful substances, also paradoxically hampers successful topical drug delivery. In the context of topical drug delivery involving the cutaneous membrane, the presence of the stratum corneum which is impermeable to water-soluble drugs and macromolecules, is one such barrier [56]. The stratum corneum is the outermost of the five layers making up the epidermis layer, which in turn is the outermost of the three main skin layers (the other two being the dermis layer directly underneath the epidermis and the subcutaneous fat tissue which seals the base of the skin) [56]. It is the respective arrangement of the corneocytes and the lipids into a “brick-and-mortar” configuration that confers such a layer its impermeability to not only water-soluble molecules but also macromolecules larger than 500 Da [44, 56].

## Conclusion

3D Printing offers a whole new platform for manufacturing in the pharmaceutical industry. It brings us a step closer in achieving personalized medicine. Pharmacies and pharmaceutical companies could be equipped with the facilities to make custom formulations to meet the needs of the patient. Polypharmacy could be overcome by combining various medications into one pill thereby improving compliance and therapeutic outcome. Chronotherapeutics, the branch of medicine aiming to maximize therapeutic efficacy whilst minimizing side effects by synchronizing drug delivery with the body’s circadian rhythm could be achieved by employing 3D fabrication [57]. From a medical perspective, surgical implants could be custom made for patients for better fit. These implants could be loaded with desired drugs to aid post-operative recovery. For instance, it has recently been reported that a drug-loaded arrowhead array device can be 3D printed using continuous liquid interface production for intraoperative implantation right after the surgical removal of tumours to deliver a combination of paclitaxel and cisplatin for the elimination of residual tumour cells at the surgical bed [58].

Thermolabile drugs cannot be used in FDM as the process involves heating the material to very high temperatures thereby limiting its use. Drug molecules can also be degraded by UV radiation that is commonly used as the polymerizing radiation in printings such as vat photopolymerization. Many 3D printed structures are porous and a batch-to-batch variation in large scale production could be a potential issue. Most of the reviewed formulations were fabricated in a small-scale setting. Scaling up to industrial level production could introduce unforeseen issues in the manufacturing.

Although 3D printing has been around for a few decades now, there is a constant need for new and improved materials that can be employed in the additive manufacturing process. Various promising research are being carried out to fabricate additive manufacturing materials suitable for pharmaceutical and biomedical use. Literature is very limited for 3D printed ocular drug delivery devices, which could be overcome with the advent of new and improved, biocompatible 3D printing materials.

From a regulatory perspective, this is a brand-new field. Pharmacopeia around the world will need to adapt and update quality control protocols and standards for the various formulations. With more sophisticated manufacturing processes and advanced materials being introduced, bench to bedside could become a reality sooner rather than later.

## Figures and Tables

**Table:** 

**3D**	Three Dimensional
**4-ASA**	4-Amino salicylic acid
**ABS**	Acrylonitrile butadiene styrene
**API**	Active Pharmaceutical Ingredient
**ASA**	Acetylsalicylic acid
**CAD**	Computer-aided design
**CIJ**	Continuous Inkjet
**CNC**	Cellulose nanocrystals
**CPT-NH**	Cryptotanshinone-loaded niosomal hydrogels
**DLP**	Digital light processing
**DMD**	Digital micromirror device
**DOD**	Drop-on-Demand
**FDM**	Fused deposition modelling
**GP120**	enveloped glycoprotein GP120
**HCQ**	Hydroxychloroquine
**HME**	Hot-melt extrusion
**HPMC**	Hydroxypropyl methylcellulose
**HPV**	Human Papilloma Virus
**HUVEC**	Human umbilical vein endothelial cells
**IgG**	immunoglobulin G
**IVR**	Intravaginal rings
**LDM**	low-temperature deposition modelling
**LED**	Light emitting diode
**MNA**	miniaturized needle arrays
**MR**	Modified Release
**NO**	Nitric oxide
**PAA**	poly (acrylic acid)
**PBF**	Powder bed fusion
**PCL**	Polycaprolactone
**PDMS**	Polydimethylsiloxane
**PEG**	polyethylene glycol
**PEGDA**	poly (ethylene glycol) diacrylate
**PLA**	polylactic acid
**PVA**	Poly (vinyl alcohol)
**PVP**	polyvinyl pyrrolidine
**SEBM**	selective electron beam melting
**SLA**	Stereolithography
**STL**	Standard Tesselation Language
**SLS**	Selective laser sintering
**TPU**	Thermoplastic polyurethane
**U.S. FDA**	United States Food and Drug Administration
**UV**	Ultraviolet
**VEGF**	Vascular endothelial growth factor
**VPP**	Vat photopolymerization
